# Decadal decline in maternal body condition of a Southern Ocean capital breeder

**DOI:** 10.1038/s41598-023-30238-2

**Published:** 2023-02-24

**Authors:** Els Vermeulen, Terriann Thavar, Maria Glarou, Andre Ganswindt, Fredrik Christiansen

**Affiliations:** 1 Mammal Research Institute, University of Pretoria, Pretoria, South Africa; 2 Húsavík Research Centre, University of Iceland, 640 Húsavík, Iceland; 3 Marine Mammal Research, Department of Ecoscience, Aarhus University, 4000 Roskilde, Denmark

**Keywords:** Bioenergetics, Ecology, Zoology, Ecology

## Abstract

The changing physical properties of the Southern Ocean are known to impact the recruitment and survival of Antarctic krill (*Euphausia superba*). For oceanic krill predators, the resulting reduced energy intake may lead to population-level effects likely preceded by an alteration in the animals’ body condition. This is especially true for capital breeders that rely on stored energy for successful reproduction. One such Southern Ocean capital breeder, the southern right whale (*Eubalaena australis*), has been monitored over the past 43 years in their South African wintering ground. Changes in the population have been documented in the past decade, including a decreased reproductive rate and a shift in foraging strategy. To evaluate if a reduced foraging success is an underlying factor, we assessed the temporal variation in morphological body condition through aerial photogrammetry. Results showed a 23% reduction in maternal body condition, potentially contributing to the decreased reproductive rate of the population. To the best of our knowledge, this is the first study to quantify a decadal reduction in the body condition of a capital breeder dependent on Southern Ocean productivity. Understanding the bioenergetic consequences of environmental change is vital to predicting species’ resilience to climate change.

## Introduction

The physical properties of the global ocean are changing rapidly under various anthropogenic impacts^1^. Critically in the Southern Ocean, survival and recruitment of Antarctic krill (*Euphausia superba*), a key prey species, are known to be gravely impacted by such physical changes, with evidence of southward contraction in their range and radical decreases in density^2,3^. Additionally being the target species of the largest volume fishery of the Southern Ocean^4^, such changes are predicted to have detrimental consequences for various top predators, by negatively affecting their vital rates (such as survival and reproduction) and population dynamics^5–9^. Considering these population-level changes often have the underlying cause of a reduced energy intake, they are likely preceded by changes in the body condition of the animals. This will be especially true in capital breeding species, that rely heavily on stored energy reserves for successful reproduction.

The body condition of an animal usually refers to its energetic state and relative amount of body reserves^10–12^. In an ecological context, indices of body condition are frequently used to measure an individual’s fitness related to survival and several important life events, such as migration and reproduction^11^. The importance of adequate body condition in successful mammalian reproduction is well established^13–16^. In baleen whales, female body condition plays a permissive role in the initiation of the reproductive cycle^17^, and determines the success of gestation^18^ and lactation^19^. Additionally, as migratory species, pregnant female baleen whales have the energetic cost associated with their long-distance migration from high-latitude feeding grounds to low-latitude calving grounds to increase the chances of calf survival after birth^20,21^. This also means that a female must synchronize her migration cycle with her reproductive cycle, which in turn has led to extremely high offspring growth rates in many baleen whale species^19,22^. It is thus clear that female baleen whales have extremely high energetic costs associated with reproduction, and that their body condition is critical for their reproductive success and therefore population growth. Unsurprisingly, the energetic cost of reproduction is known to be the most prominent trade-off in the life history of most species when sufficient energy cannot be acquired^23^. This also holds for marine mammals, with multiple studies showing clear links between reduced prey availability and/or maternal body condition and reproductive output^13,14,16,19,24–28^.

As a capital breeder, southern right whales (*Eubalaena australis*) usually forage at higher latitudes during the austral summer months and calve and nurse at low-latitude coastal areas during the austral winter months. The population calving in the coastal waters of South Africa has been studied extensively since 1979^29^. These studies have shown a seasonal presence of mothers and calves between June and November, with a peak in birthing in August, lasting up to late October/early November^30^ The long-term sighting history dataset of individually identified parous females has enabled the estimation of vital population demographic parameters including the steady recovery of the population (at around 7% per annum) post-whaling^31^. However, the last decade has been characterised by some noteworthy changes, including a decreased coastal prevalence and an increase in calving intervals from 3- to 4- and 5-year intervals^32^ suggestive of a reduced reproductive success^33^. Furthermore, a recent study revealed a dramatic northward shift, and diversification, in the foraging strategy of South Africa’s southern right whales since the 1990s^34^. Considering the species is known to show fidelity to foraging grounds^35,36^, the authors suggested this shift is likely related to environmental changes in preferred foraging habitats^34^.

To evaluate the effects of this shift on the continued success of the population’s foraging, here we assess the variation in the body condition of parous South African southern right whale females over a similar temporal scale and discuss the possible link to the concurrent reduced reproductive success. Certainly, understanding not only the behavioural but also the bioenergetic consequences of environmental change is vital for our insight into how effectively predators may be responding to such changes, and for our ability to predict more precisely how resilient these species are in light of climate change. In light of the documented changes in foraging strategy and calving rates, we hypothesise that the body condition of southern right whales of South Africa has declined over the last decades.

## Materials and methods

### Data collection

**Figure 1:**
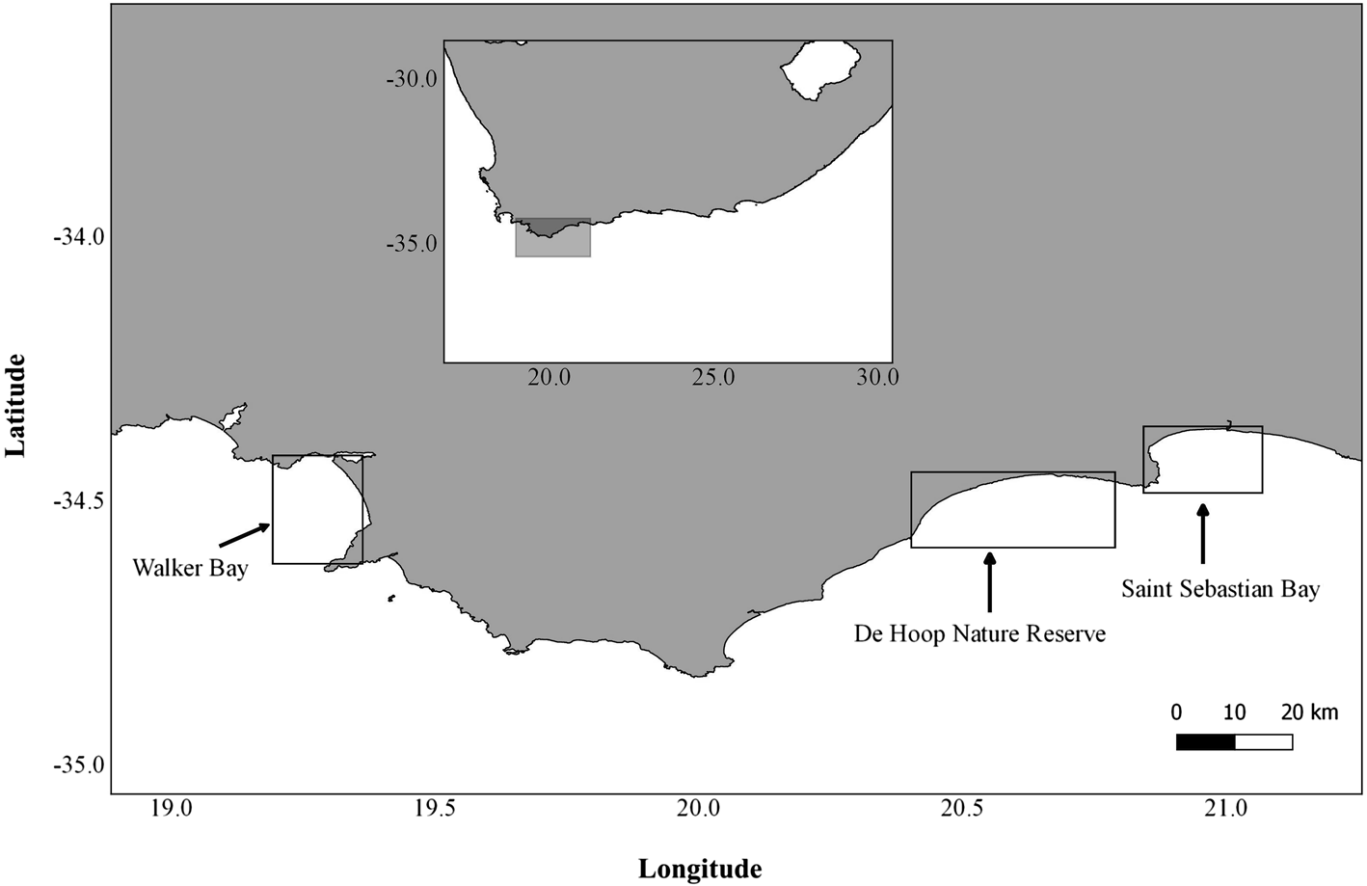
Map of study areas in South Africa. Sample collection in the Early Period took place in De Hoop Nature Reserve, whereas sampling in the Late Period took place in Saint Sebastian Bay and Walker Bay (created in QGIS 3.24.3).

Aerial photographs of southern right whale cow and calf pairs were collected in South African coastal waters from a manned aircraft in the late 1980s (1988 and 1989, hereafter referred to as the “early period”) and by unoccupied aerial vehicles (UAVs) around 2020 (2019 and 2021, hereafter referred to as “late period”). Sampling during the early period took place in De Hoop Nature Reserve in mid-October by Best and Ruther^37^ (Fig. 1). Zenithal photographs of surfacing whales were taken from a helicopter using a vertically mounted Hasselblad ELM camera with a 250 mm lens^37^. The altitude during sampling varied between 75 and 120 m. The original film negatives were scanned professionally for conversion to digital images. Sampling during the late period was conducted using a DJI Phantom 4 Pro (diameter = 350 mm, weight = 1388 g, 1″ CMOS sensor, 20MP, 5472 × 3078 pixels, 8.8 mm focal length) multirotor UAV (in 2019 and 2021) and a DJI Mavic 2 Zoom (diameter = 322 mm (unfolded), weight = 905 g, 1/2.3″ CMOS sensor, 12MP, 4000 × 3000 pixels, 24–48 mm focal length) UAV (in 2021), and took place in Walker Bay and Saint Sebastian Bay during early-September (Fig. 1). The altitude during sampling varied between 15 and 25 m. All three survey areas are known aggregation areas for mother-calf pairs along the South African coast, with movement between them^38^.

### Data processing and filtering

Following the established protocol of southern right whale photogrammetry^19^, the best photograph of each individual whale was selected and quality graded based on camera focus, body posture (horizontal roll, body arch and vertical pitch) and body shape visibility (ability to see the rostrum, fluke notch and the body contour). Each photograph was given a score from 1 (good) to 3 (poor) for each of these attributes. Those photographs which had at least one score of three were excluded for further analyses. Additionally, images with a score of two for at least two of the three categories under body posture were excluded from further analyses. Duplicate measurements of the same individuals were removed from analyses to avoid pseudo-replication, with the photograph with the highest quality being retained in the data set.

From the aerial photographs, the body length and widths (at 5% increments perpendicular to the body axis) of the whales were measured, in pixels, using a custom-written script^39^ in R version 4.0.3^40^.

### Body condition metric

Since the energy content of whales is given by their body mass^41^, we decided to use the body volume to describe the energy stores of southern right whales. This metric closely relates to body mass and incorporates the full 3D shape of the animals, in contrast to some other metrics (e.g. body area index).

As the inbuilt barometric altimeter of the UAV did not provide accurate altitude measurements, and no precise altitude data were available for the photographs collected in the early period, only relative length and width measurements (standardized against a body length of 1) were used in this study.

For each width measurement, the corresponding height measurement (dorso-ventral distance) was calculated, using a published height-width ratio of southern right whales^42^.The body volume (BV) of each whale was then estimated using an elliptical volume approach^42^.

1 $$ BV_{s,i} = BL_{i} \times 0.05 \times \mathop \smallint \limits_{0}^{1} \pi \times \frac{{W_{A,s,i} + \left( {W_{P,s,i} - W_{A,s,i} } \right) \times x}}{2} \times \frac{{H_{A,s,i} + \left( {H_{P,s,i} - H_{A,s,i} } \right) \times x}}{2}dx $$

2 $$ BV_{Total,i} = \mathop \sum \limits_{s = 1}^{20} V_{s,i} $$

where *s* is the section of the body between two adjacent width/height measurement sites (S = 20 in total), *BL*_*i*_ is the body length of whale *i* (set to 1), *W*_*A,s,i*_ and *H*_*A,s,i*_ are the anterior relative width and height measurements (expressed as proportion of BL) of body segment *s* for individual *i*, and *W*_*P,s,i*_ and *H*_*P,s,i*_ are the posterior relative width and height measurements (expressed as proportion of BL) of segment *s* for individual *i*, respectively. To account for the gradual decrease in height and width towards the end points of the animal, the segments closest to the rostrum (0–5%BL from the rostrum) and the end of the tail region (85–100%BL from the rostrum) were modelled as elliptical cones^41^.

Subsequently, the body condition (BC) was calculated using the BC formula^19^ modified for relative measurements:

3 $$ BC = \frac{{BV_{Obs} - \mu \left( {BV} \right)}}{{\mu \left( {BV} \right)}} $$

where *BV*_*Obs*_ is the observed (measured) body volume, standardized against a body length of 1, and *µ*(*BV*) is the mean body volume of the sample population, also standardized against a body length of 1. A positive BC of 0.2 means that an animal has a body volume that is 20% higher than the average of the sample population and a negative BC of − 0.2 indicates that the animal has a body volume that is 20% lower than the average individual in the sample population. A previous study demonstrated that the relative BC (volume estimates standardized against a body length of 1) of right whales is nearly identical (R^2^ = 0.999) to their absolute BC (volume estimated from absolute morphometrics)^43^. Since all measurements in our study were standardized against a body length of 1, the BC could hence be estimated without knowing the absolute length of the animals.

### Comparison of body condition between the early and late period

Linear models (LMs) were developed in R to compare the body condition (response variable) of southern right whale mothers and calves between the early and late periods (explanatory variable). Covariates included relative calf length (% of maternal length) since maternal body condition in southern right whales is known to decline as the calf grows in length through the breeding season^19,43^. Year was also included as a covariate factor to see if there was a difference in body condition within the early (between southern right whales in 1988 and 1989) and the late (between southern right whales in 2019 and 2021) periods. Due to collinearity, year and period could not be included in the same models. Day of year was also excluded as a covariate since it varied significantly between sampling years (LM: F_3,43_ = 1076.1, *p* < 0.001) and was hence correlated with both year and period. This was not an issue, since relative calf length has been shown to be a better predictor of seasonal changes in maternal body condition compared to Day of year in right whales^19,42^. Model selection was based on minimization of Akaike’s Information Criterion (AIC). In case of a ΔAIC < 2, the most parsimonious model was selected. Separate analyses were carried out for lactating females and calves. Model diagnostics tests were run to ensure that the model assumptions were met for each model. These included scatter plots of model residuals against fitted model values (to evaluate homogeneity of residuals), frequency histograms of model residuals (to examine normality of residuals), leverage scores (to identify influential data points) and Cook’s distance (to identify outliers). All model assumptions were met.

### Accounting for measurement errors

To assure that measurement errors inherent to the sampling method (aerial photogrammetry) did not confound our comparative analyses, we conducted a sensitivity analysis to quantify the measurement errors on the model parameter estimates. The coefficient of variation (CV) in body width measurements associated with the different width measurability scores (the ability to accurately see the body contour of the whales) was estimated to be 2.11 and 2.31% for quality 1 and 2, respectively^19^. The mean measurement error between photographs of the same whale within the same day was 4.75% (SD = 3.674) for lactating females and 3.11% (SD = 2.206) for calves^19^. By randomly allocating new body width values to the measured whales (1,000 times), based on these error distributions, and refitting the best fitting model for lactating females and calves, we could obtain a distribution of parameter values for the best fitting models and compare it to the original model parameter estimates.

## Results

### Sampling effort

Data were collected on the 15th and 13th of October in 1988 and 1989, respectively, between the 3rd and 15th of September (7 sampling days) in 2019, and between the 2nd and 8th of September (3 sampling days) 2021 (Table 1). A total of 187 aerial images of southern right whale mothers and calves were obtained during the early period and 120 during the late period. After data filtering (based on picture quality and duplicate measurements), 46 aerial images (20 lactating females and 26 calves) remained from the early period and 75 images (27 lactating females and 48 calves) from the late period (Table 1).

**Table 1:** Composition of the southern right whales sampled in South Africa by aerial photogrammetry by year and reproductive class.

Year	Period	Dates		Sampling effort		Number of individuals measured	
		Start	End	Duration	Days	Calves	Lactating females
1988	Early	Oct-15	Oct-15	1	1	13	10
1989	Early	Oct-13	Oct-13	1	1	13	10
2019	Late	Sep-3	Sep-15	13	7	39	20
2021	Late	Sep-2	Sep-8	7	3	9	7

N = 121 measured whales.

### Comparison of body condition between the early and late period

**Figure 2:**
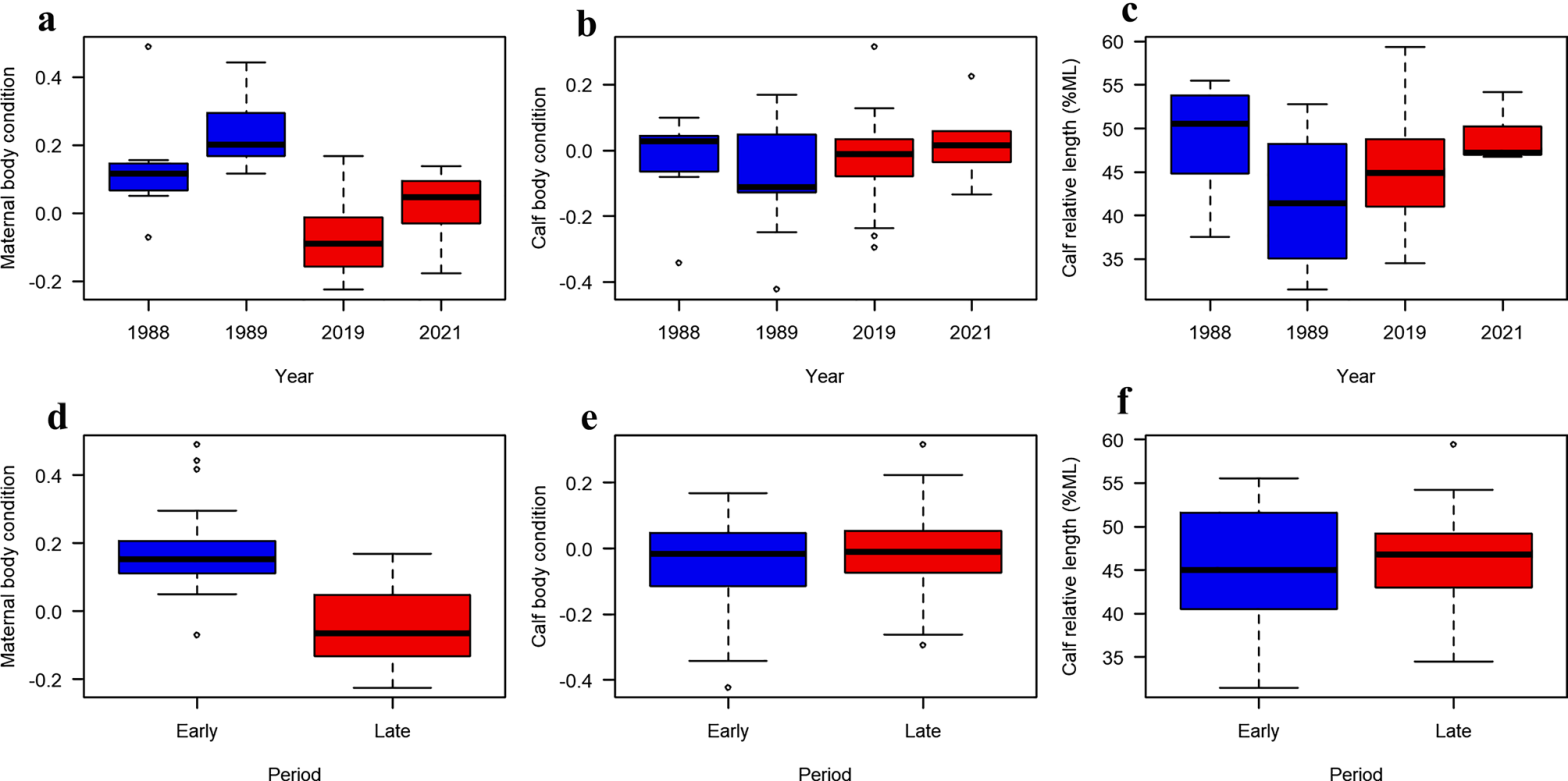
Boxplots showing the observed variation in body condition and length. (**a** and **d**) show the observed variation in maternal body condition, whereas (**b** and **e**) show the variation in calf body condition, and (**c** and **f**) show the variation in calf relative body length of southern right whales for the different sampling years (top row) and periods (bottom row). The blue and red colours indicate measurements taken during the early and late periods, respectively. *ML* maternal body length. For sample sizes, see Table 1.

There was considerable variation in body condition of lactating females and calves, as well as relative calf-length, between study years and periods (Fig. 2). For lactating females, the best-fitting model included year as a single explanatory variable (Model 3 in Table 2).

**Table 2:** Linear model selection results based on minimization of Akaike’s information criterion (AIC) for southern right whale maternal body condition (MBC).

Model	Variables	F	*df* (among)	*df* (within)	*P*	R^2^	AIC	ΔAIC
1	MBC ~ 1 (null model)	*–*	0	46	*–*	0.00	− 31.0	31.0
2	MBC ~ Period	39.3	1	45	< 0.001	0.47	− 58.5	3.5
3	MBC ~ Year†	17.1	3	43	< 0.001	0.54	− 61.9	0.0
4	MBC ~ CL	0.7	1	45	0.405	0.02	− 29.7	32.2
5	MBC ~ Period + CL	19.7	2	44	< 0.001	0.47	− 57.0	4.9
6	MBC ~ Period × CL	16.5	3	43	< 0.001	0.53	− 60.9	1.0
7	MBC ~ Year + CL	12.6	4	42	0.001	0.55	− 60.1	1.9
8	MBC ~ Year × CL	7.5	7	39	0.001	0.57	− 57.1	4.8

*CL* calf relative length (% maternal length).

^†^The most parsimonious model (Model 3).

**Figure 3:**
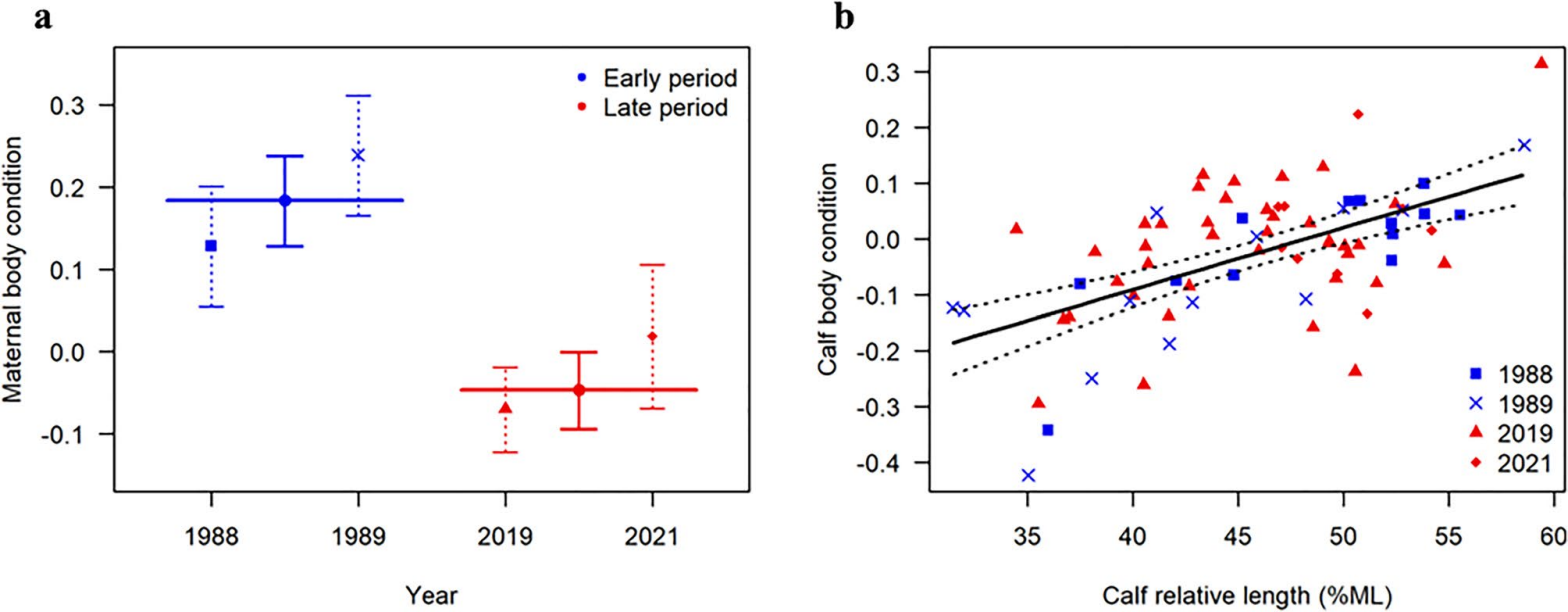
(**a**) Effects of year and period (see colour key) on maternal body condition in South Africa’s southern right whales. The points show the predicted mean body condition values from the best-fitting model for year (Model 3 in Table 2) and period (Model 2 in Table 2). The error bars show the 95% confidence intervals. (**b**) Calf body condition as a function of calf relative body length (% of maternal length, ML). The solid black line shows the predicted mean values of the best-fitting model (Model 4 in Table 3). The dotted black lines show the 95% confidence intervals.

The body condition of lactating females varied significantly between years (LM: F_3,43_ = 17.1, *p* < 0.001), with the model explaining 54.5% (R^2^) of the variance in the data. The body condition of lactating females was significantly higher in 1988 (mean = 0.128, SE = 0.0372) and 1989 (mean = 0.239, SE = 0.0372) compared to 2019 (mean = − 0.070, SE = 0.0263) and 2021 (mean = 0.019, SE = 0.0444) (Fig. 3a). Further, the body condition in 1989 was 0.111 (SE = 0.0526) higher than in 1988 (Fig. 3a). There was no significant difference in body condition between 2019 and 2021 (Fig. 3a). If replacing Year with Period (Model 2 in Table 2), there was a significant difference in the body condition of lactating females between the early and late period (LM: F_1,45_ = 39.3, *p* < 0.001), with females being 23% (SE = 3.68) fatter in the early period (mean = 0.184, SE = 0.0279) compared the late period (mean = − 0.047, SE = 0.0240) (Fig. 3a). The model explained 46.6% (R^2^) of the variance in the data. The results of the model selection showed that adding calf length to the model did not significantly improve model fit (ΔAIC < 2), and the model that included year as the only explanatory variable was the most parsimonious (see Table 2). Hence, even though the range of calf length varied between years (LM: F_3,43_ = 3.27, *p* = 0.030) (Fig. 2c), which could explain the marginally better fit of the LM including period × calf length (Model 6 in Table 2) compared to the LM including only period as a covariate (Model 2 in Table 2) , the magnitude was not sufficient to affect the BC between years, and hence was not a confounding factor.

For calves, period and year had no significant effect on their body condition, and the best-fitting model only included calf relative body length as a covariate (Model 4 in Table 3). Calf body condition increased significantly with relative body length (LM: F_1,72_ = 34.6, *p* < 0.001), at a rate of 0.011 (SE = 0.0019) for every % increase in relative calf length (Fig. 3b). The model explained 32.5% (R^2^) of the variance in the data.

**Table 3:** Linear model selection results based on minimization of Akaike’s information criterion (AIC) for southern right whale calf body condition (CBC).

Model	Variables	F	*df* (among)	*df* (within)	*P*	R^2^	AIC	ΔAIC
1	CBC ~ 1 (null model)	*–*	0	73	*–*	0.00	− 98.0	27.5
2	CBC ~ Period	1.7	1	72	0.199	0.02	− 97.7	27.8
3	CBC ~ Year	1.5	3	70	0.213	0.06	− 96.8	28.8
4	CBC ~ CL^†^	34.6	1	72	< 0.001	0.32	− 125.1	0.4
5	CBC ~ Period + CL	18.5	2	71	< 0.001	0.34	− 125.1	0.5
6	CBC ~ Period × CL	13.4	3	70	< 0.001	0.36	− 125.5	0.0
7	CBC ~ Year + CL	9.1	4	69	< 0.001	0.34	− 121.3	4.3
8	CBC ~ Year × CL	5.5	7	66	< 0.001	0.37	− 117.9	7.7

*CL* calf relative length (% maternal length).

^†^The most parsimonious model (Model 4).

### Effect of measurement errors

**Figure 4:**
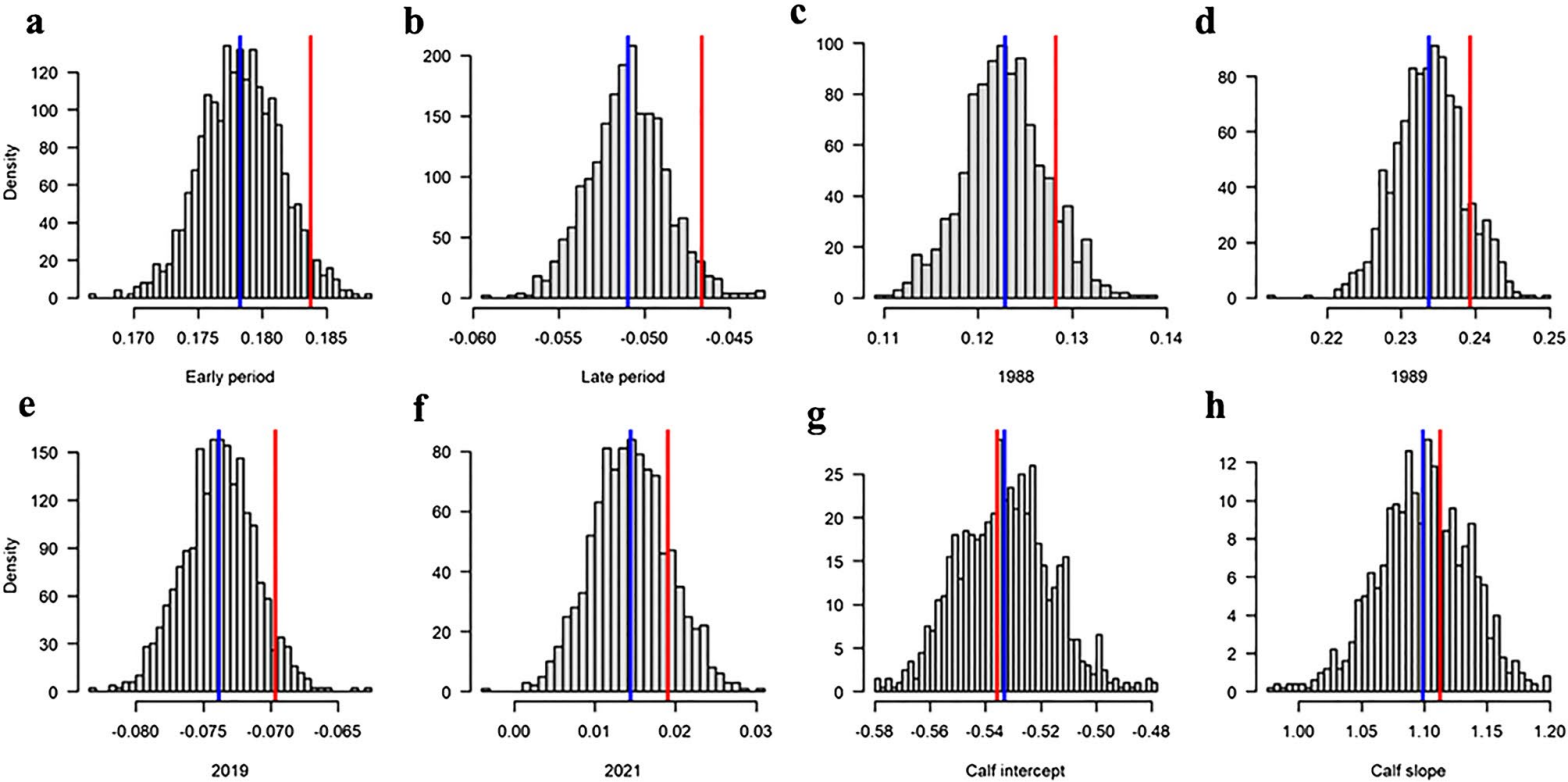
Output of the sensitivity analysis of measurement errors (width measurability errors and between picture measurement errors), showing the density distribution of the model parameter values for (**a**, **b**) the effect of period on maternal body condition (Model 2 in Table 2), (**c**–**f**) the effect of year on maternal body condition (Model 3 in Table 2), and (**g**, **h**) the intercept and slope parameter of the linear relationship between calf body condition and calf relative body length (Model 4 in Table 3). The vertical blue lines show the mean parameter values of the sensitivity analysis. The vertical red lines show the original parameter values.

The sensitivity analysis showed that there was no overlap of the density distributions of the predicted body condition values of lactating females between the early and late study period (Fig. 4a,b, Model 2 in Table 2), and no overlap between years (Fig. 4c–f, Model 3 in Table 2). For calves, the density distributions of the intercept and slope parameters of the LM between calf body condition and calf relative body length showed no overlap with zero (Fig. 4g,h, Model 4 in Table 3). The output of the sensitivity analysis hence showed that all the model parameters were robust to measurement errors.

## Discussion

Results of the present study demonstrate a 23% reduction in maternal body condition of South African southern right whales since the late 1980s, which is in agreement with our hypothesis. To put this in perspective, female southern right whales lose up to 25% of their body condition in the first three months of lactation^19^. Concurrent to this decadal decrease, stable isotope data from skin samples taken in the early 1990s and the 2010s revealed a northward shift in foraging location, and an apparent diversification in targeted prey over the same time period, suggestive of changes in previously preferred foraging habitat and/or prey^34^. Considering baleen whale body condition can be directly linked to prey abundance^44^ these data are clearly indicative of a drastic reduction in foraging success and/or reduced prey availability.

Prey availability for oceanic top predators is strongly linked with climatic and oceanographic processes, and the impact of changes in these processes on the trophic ecology of oceanic predators are well documented^6,8,9,25,27,45–52^. Based on whaling data, Antarctic krill constitutes the key food source for southern right whales when feeding south of 50°S^53^. However, multiple studies have shown a strong inverse relation between krill abundance, rising sea surface temperatures (hereafter ‘SSTs’) and sea-ice loss due to climate change^54,55^, which decreases prey availability for many species throughout the food web. Antarctic krill are also known to be transported by frontal systems (they are, however, also capable of active vertical and horizontal migrations^56^), sustaining populations outside of the main spawning areas. However, various studies indicate displacement of such frontal systems of the Antarctic Circumpolar Current due to climate change^45^, possibly another contributing factor to decreased food availability for many krill-dependent species, including those outside main spawning areas. Ultimately, the impacts of climate change on krill are complex and multifaceted. Furthermore, recent changes to key oceanographic parameters such as SST, productivity, wind speed, mixed layer depth, sea-ice duration and frontal positions are not uniform across the Southern Ocean, causing substantial spatial and seasonal variation^50,57^.

When foraging between 50° and 40°S, southern right whales seem to also increase the intake of copepods^53^, which are much smaller in size and biomass than krill^58^. However, some studies suggest that the nutritional content (caloric value) in copepods has changed over time due to climate change and sea ice loss^59–62^. Considering the reported north-ward shift in the foraging location of this population^34^, it is possible that a shift in diet combined with a reduction in the nutritional value of southern right whale prey may have played a role in the reduction of South African southern right whale body condition in recent years. This hypothesis has also been indirectly eluded to in a previous study investigating southern right whale calf mortality in Argentina^63^, and warrants further investigation.

Body condition, and thus foraging success, influences reproduction in most mammals^64^, as it is crucial in all parts of reproduction including ovulation, gestation and lactation. This is also the case in marine mammals and various studies have shown such a direct relationship to exist. For example, a reduced prey abundance correlated with reduced fecundity in killer whales (*Orcinus orca*)^65^, and a decreased rate of pregnancy in North Atlantic fin whales (*Balaenoptera physalus*) was related to prey limitation mediated through body condition^28^. Furthermore, the calving rates of North Atlantic right whales (*Eubalaena glacialis*) were correlated with various lags to the abundance of copepods (*Calanus finmarchicus*)^66^, and so poor body condition was indicated as a cause for the observed long calving intervals in the species^43^. Such a direct relationship has also been found for southern right whales in the Southwest Atlantic, in which the reproductive success was shown to be dependent on prey availability and thus foraging success^25,27^. This reliance on stored energy is furthermore illustrated by the fact that southern right whale mothers lose up to 25% of their body condition during the first few months of lactation^19^. It is therefore clear that adequate nutrition and thus foraging success is critical for the reproductive success of southern right whales and ultimately, ensure their continued recovery post-whaling. It is perhaps unsurprising then, that the reduced body condition of South African southern right whales falls within a period of increased calving intervals observed in the population^67^. Indeed, although 3-year calving cycles are usually predominant in the species^31^, including one year of gestation, one year of lactation and one year of rest (to allow the female to recover and accumulate enough energy reserves to support the following pregnancy), this ceased to be the case in the South African population in the past decade when a drastic increase in 4- and 5-year calving intervals became apparent^32^. These 4- and 5-year calving intervals may imply either additional resting years (to build up fat reserves) between successive calving events or a loss of a fetus^33^.

Combined, available evidence are strongly indicative of a reduced body condition of female southern right whales to an extent where it affects their calving rate. Furthermore, maternal body condition does not only affect calving rates but also calf and juvenile growth rate^19,68^ as well as the age of first parturition^69^, ultimately affecting population growth. Although a reduced calf condition is not visible in the data, the present findings raise serious concern for the continued reproductive success and population recovery post-whaling of the South African population of southern right whales. Continued research into these different facets of reproduction, including for the various demographic groups in the population, are important to fully understand the effect of the reduced maternal body condition on the population.

In the past decade, research on the South African southern right whale population has shown evidence of drastic population-level changes likely as a response to environmental changes in their foraging habitat^34,67^. Such changes are not unique to South African waters, as in recent years, fluctuations in southern right whale counts^32^, elongation of calving intervals^32,70,71^ and increased calf mortality^71,72^ have been observed across several wintering grounds, with (at least) short-term effects on the population growth rate^73,74^. While certain discrepancies in changes between the different breeding populations exist, they all seem directly or indirectly related to altered prey availability^25,27,34,75^. The species can therefore represent a sentinel for environmental change in their South Ocean foraging grounds.

As the global ocean changes in response to a host of anthropogenic drivers^1^, top ocean predators will experience climate change directly through physiology or indirectly through alterations in food web dynamics^76^. Arguably, impacts on food web dynamics can be deemed most critical for predators such as baleen whales, as they may be more flexible in their physiological and behavioural responses to increasing SST^77^. In fact, various studies have indicated that the predicted changes for the Southern Ocean, driven by global climate change, should be of great concern precisely because it holds crucial nursery habitats and foraging grounds for krill^47,50,78^, an important food source for many marine top predators^27,51,53,79,80^. To the best of our knowledge, our study is the first to quantify a temporal reduction in the body condition of a baleen whale dependent on Southern Ocean productivity, clearly indicating a reduced foraging success over time. Although the environmental changes that may be driving this change remain to be determined, they clearly result in either a decreased prey availability and/or a decreased quality of prey for these marine predators. Unsurprisingly, the size, complexity, and relative difficulty of attaining long-term measurements in the Southern Ocean make it difficult to develop a full spatial understanding of oceanographic variation in the Southern Ocean under climate change^81^. Therefore, future studies assessing the changes occurring in oceanographic conditions in the Southern Ocean, known to be important in driving suitable southern right whale feeding habitat^82^, could help to alleviate this considerable knowledge gap.

## Data Availability

The data that support the findings of this study are available from the corresponding author upon reasonable request.
